# Discovery of Two Mycoviruses by High-Throughput Sequencing and Assembly of Mycovirus-Derived Small Silencing RNAs From a Hypovirulent Strain of *Sclerotinia sclerotiorum*

**DOI:** 10.3389/fmicb.2019.01415

**Published:** 2019-07-02

**Authors:** Qianqian Wang, Shufen Cheng, Xueqiong Xiao, Jiasen Cheng, Yanping Fu, Tao Chen, Daohong Jiang, Jiatao Xie

**Affiliations:** ^1^State Key Laboratory of Agricultural Microbiology, Huazhong Agricultural University, Wuhan, China; ^2^Hubei Key Laboratory of Plant Pathology, College of Plant Science and Technology, Huazhong Agricultural University, Wuhan, China

**Keywords:** *Sclerotinia sclerotiorum*, mycovirus, botybirnavirus, *Tymoviridae*, high-throughput sequencing, small RNA

## Abstract

*Sclerotinia sclerotiorum*, an important phytopathogenic fungus, harbors rich diversity of mycoviruses. Lately, more mycoviruses can be successfully and accurately discovered by deep sequencing, especially those that could not be detected by traditional double-stranded RNA (dsRNA) extraction. Previously, we reported that the hypovirulent *S. sclerotiorum* strain SZ-150 is coinfected by Sclerotinia sclerotiorum hypovirus 1 (SsHV1) and its related satellite RNA. Here, aside from SsHV1, we detected two other mycoviruses, Sclerotinia sclerotiorum botybirnavirus 3 (SsBV3/SZ-150) and Sclerotinia sclerotiorum mycotymovirus 1 (SsMTV1/SZ-150), coinfecting strain SZ-150, by deep sequencing and assembly of mycovirus-derived small RNAs and determined their full-length genomes. The genome of SsBV3/SZ-150 was found to be composed of two linear dsRNA segments, 6,212, and 5,880 bp in size, respectively. Each dsRNA segment of SsBV3/SZ-150 contains a large open reading frame (ORF) encoding RNA-dependent RNA polymerase (RdRp) and a hypothetical protein. The whole genome of SsBV3/SZ-150 shares more than 95% sequence identity with Botrytis porri botybirnavirus 1 (BpBV1) at the nucleotide (nt) or amino acid level. Thus, SsBV3/SZ-150 was assumed to be a strain of BpBV1. The genome of SsMTV1/SZ-150 consists of 6,391 nt excluding the poly(A) tail. SsMTV1/SZ-150 was predicted to contain a large ORF that encodes a putative replication-associated polyprotein (RP) with three conserved domains of viral RNA methyltransferase, viral RNA helicase, and RdRp. Phylogenetic analyses suggest that SsMTV1/SZ-150 is related, albeit distantly, to members of the family *Tymoviridae*. Analysis of the small RNAs derived from SsBV3/SZ-150 and SsMTV1/SZ-150 revealed that small-RNA lengths mainly range from 20 to 24 nt, with a peak at 22 nt, and the most abundant 5′-terminal nucleotide is uridine, suggesting that the Dicer 2 and Argonaute 1, two key components in the RNA inference pathway, may play important roles in the resistance to mycoviral infection in *S. sclerotiorum.* Neither SsBV3/SZ-150 nor SsMTV1/SZ-150 is a causal agent of hypovirulence in strain SZ-150.

## Introduction

Mycoviruses (or fungal viruses) are viruses that infect fungi and Oomycete species and depend on their hosts for replication. Since the first mycovirus was reported in 1962 (Hollings, 1962), mycoviruses have been identified in all major taxonomic groups of fungi and have shown remarkable diversity (Ghabrial and Suzuki, 2009; Pearson et al., 2009; Ghabrial et al., 2015). For defense against mycovirus infection, fungi have evolved antiviral defense mechanisms of RNA silencing, which is similar to that of other eukaryote hosts including plants and animals (Torres-Martinez and Ruiz-Vazquez, 2017; Guo et al., 2019). RNA virus infection in fungi induces production of virus-derived small RNAs (vsRNAs) that have specific function in the RNA-silencing process (Wang et al., 2016; Yaegashi et al., 2016; Donaire and Ayllon, 2017; Yu et al., 2018). These vsRNAs share features with host endogenous small interfering RNAs (siRNAs) and can potentially mediate RNA silencing pathways or can regulate viral replication. Therefore, deep sequencing of small RNAs from virus-infected fungi is an efficient strategy to discover and identify mycoviruses, especially latent ones.

*Sclerotinia sclerotiorum*, a destructive Ascomycota fungus, is capable of attacking more than 400 species of plants, and cause great losses in a wide variety of crops throughout the world (Bolton et al., 2006). Since the first double-stranded RNA (dsRNA) element was found in a hypovirulent *S. sclerotiorum* strain 91 (Boland, 1992), this fungus is increasingly recognized to harbor a great variety of mycoviruses, including dsRNA viruses, single-stranded RNA (ssRNA) viruses, and one single-stranded circular DNA virus. Some of those mycoviruses confer hypovirulence to their hosts and show a great potential for virocontrol (Xie and Jiang, 2014). At present, with development of the high-throughput sequencing technology, novel mycoviruses that are difficult to find by conventional detection methods, such as dsRNA extraction, can be discovered in *S. sclerotiorum* (Marzano et al., 2016; Mu et al., 2017). These discovered *S. sclerotiorum* mycoviruses not only enrich known virus diversity but also supply new knowledge about viral evolution and its coevolution with the host (Jiang et al., 2013; Marzano et al., 2016).

The order *Tymovirales* was first established in 2004 and is currently composed of five families: *Alphaflexiviridae*, *Betaflexiviridae*, *Gammaflexiviridae*, *Deltaflexiviridae*, and *Tymoviridae*^1^. Members of this order possess an ssRNA genome from 5.9 to 9.0 kb in length, which is mostly polyadenylated. The largest protein, i.e., replication-associated polyprotein (RP), with molecular mass approximately 150–250 kDa, is encoded in all the members of the order *Tymovirales*, and the RP protein usually consists of a set of conserved functional domains (King et al., 2011). The members of *Tymovirales* show great diversity in virion morphology. The members of the families *Alphaflexiviridae*, *Betaflexiviridae*, and *Gammaflexiviridae* have virions in the form of flexuous filaments, whereas viruses in the family *Tymoviridae* have non-enveloped isometric particles (Adams et al., 2011b). The viruses belonging to *Tymovirales* also have diverse host ranges. Members of *Betaflexiviridae* and *Tymoviridae* are plant viruses (Adams et al., 2011b), a single member of *Gammaflexiviridae* is reported only from a filamentous fungus (Howitt et al., 2001; Adams et al., 2011b), whereas members of *Alphaflexiviridae* infect both plants and fungi (Adams et al., 2011a).

Botybirnaviruses are a group of bipartite viruses with dsRNA genomes of 5,800–6,500 bp for each segment. The first botybirnavirus, Botrytis porri botybirnavirus 1 (BpBV1), has been discovered in the phytopathogenic fungus *B. porri* and is responsible for hypovirulent traits (Wu et al., 2012). Two botybirnaviruses have been subsequently found in *S. sclerotiorum* (Liu et al., 2015; Ran et al., 2016). The botybirnavirus infection is associated with the hypovirulence of *S. sclerotiorum* as well. Recently, more candidate members of the genus of Botybirnavirus were detected in the fungi of Ascomycota, e.g., fungi of genera *Alternaria* and *Bipolaris* (Xiang et al., 2017; Wang et al., 2018). With the development of sequencing technology, more botybirnavirus-like sequences have also been identified during the metatranscriptomic analysis, e.g., the finding of botybirnavirus-like sequences in the metatranscriptomics survey of soybean phyllosphere phytobiomes (Marzano and Domier, 2016). Therefore, botybirnaviruses may have a wide distribution among different fungal groups.

Previously, we have reported three dsRNA segments, two similarly, sized at 9.5 kbp and a third one of approximately 3.6 kbp. One of the two large dsRNA segments, the replication form of Sclerotinia sclerotiorum hypovirus 1 (SsHV1/SZ-150), has been characterized (Xie et al., 2011). SsHV1/SZ-150 is closely related to CHV3/GH2 and CHV4/SR2 within *Hypoviridae*, whereas the 3.6-kbp dsRNA segment was recognized to be the satellite-like RNA (SatH) of SsHV1/SZ-150. The partial cDNA sequence of the other large dsRNA segment (the genome of the previously named Sclerotinia sclerotiorum RNA virus 1, SsRV1/SZ-150) was determined and putatively assigned to a plant virus (Xie et al., 2011). In the present study, we obtained sRNA from the hypovirulent strain SZ-150 of *S. sclerotiorum* by deep sequencing and discovered two other mycoviruses, Sclerotinia sclerotiorum botybirnavirus 3 (SsBV3/SZ-150, originally named SsRV1/SZ-150) and Sclerotinia sclerotiorum mycotymovirus 1 (SsMTV1/SZ-150) aside from SsHV1 and SatH. Combined with bioinformatics analysis and rapid amplification of cDNA end (RACE), full-length genomes of these two mycoviruses were determined *via* assembly of myco vsRNAs and were characterized. The assay of biological features on individual strains with different virus combination confirmed that neither SsBV3/SZ-150 nor SsMTV1/SZ-150 is a causal agent of hypovirulence of strain SZ-150.

## Results

### Assembled Small RNAs and Identification of Two New Mycoviruses

Large-scale sequencing of sRNAs has been used for virus identification in insects, plants, and fungi (Kreuze et al., 2009; Wu et al., 2010; Vainio et al., 2015). To test whether the strain SZ-150 is infected by other new mycoviruses aside form SsHV1/SZ-150 and its SatH, the total RNA from the strain SZ-150 was prepared for deep sequencing of sRNAs. After removing of the adaptors and filtering out low-quality tags, the total number of reads produced by the Illumina sequencer was 12,999,900 [read length ranging from 18 to 30 nucleotides (nts)]. To obtain more information about mycovirus-related sequences, these sRNAs were first matched against the genome sequences of *S. sclerotiorum*, SsHV1/SZ-150 and SatH/SZ-150. The unmatched sRNAs were then assembled into longer contiguous sequences (contigs) before they were used for sequence similarity searches in reference databases. The newly assembled contigs could be subdivided into two groups based on BLASTX against NCBI GenBank database. One group of contigs contained sequences sharing a higher identity (95–100%) with BpBV1/GarlicBc-72, and those contigs were presumed to represent the genome of a putative mycovirus, namely, SsBV3/SZ-150 (accession numbers MK530703 and MK530704) to distinguish it from two botybirnaviruses previously found in *S. sclerotiorum* (Liu et al., 2015; Ran et al., 2016). The second group of sequences revealed that proteins encoded by two contigs shared limited sequence identity (27–39%) with members of the family *Tymoviridae*, suggesting that they represent the genome of a new virus. We tentatively named this virus SsMTV1/SZ-150 (accession number MK530705). Thus, SZ-150 is coinfected with three mycoviruses: two (+)ssRNA viruses (SsHV1 and SsMTV1) and one virus with dsRNA genome (SsBV3), along with a SatH of SsHV1/SZ-150.

### Genome Organization and Characterization of SsBV3/SZ-150 and SsMTV1/SZ-150

As a result of a combination of the assembled vsRNA contigs and RACE experiments, the complete genomes of SsBV3/SZ-150 and SsMTV1/SZ-150 were determined. Genomes of both viruses were re-sequenced *via* RT-PCR with specific primers based on the assembled sequence to ensure maximum sequence accuracy (Supplementary Table S2).

#### Characterization of SsBV3/SZ-150

**FIGURE 1:**
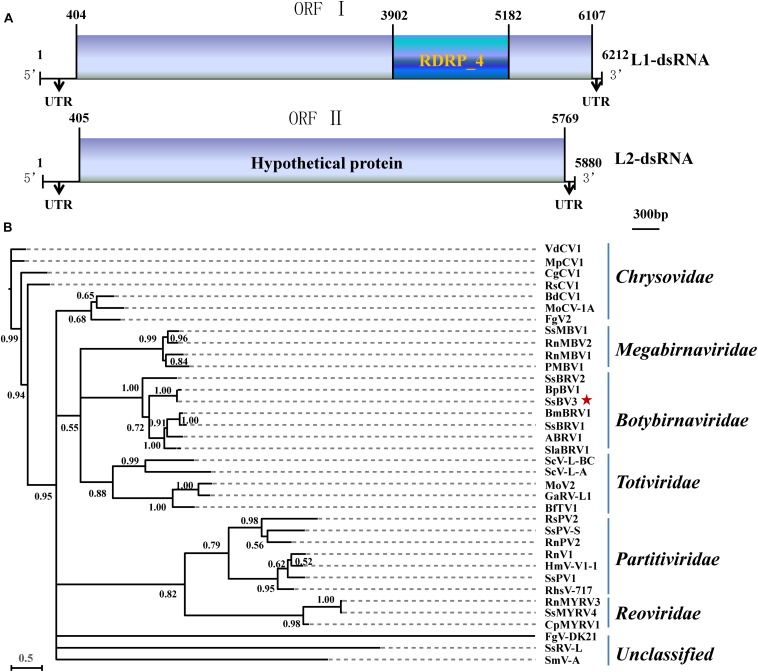
Schematic diagram and phylogenetic analysis of Sclerotinia sclerotiorum botybirnavirus 3 (SsBV3/SZ-150). **(A)** Genomic organization of SsBV3/SZ-150. The 5′- and 3′-UTRs (line) and the ORF (box) are displayed. The blue box indicates a conserved RdRp domain. The scale bar corresponds to a length of 300 bp. **(B)** A Bayesian phylogenetic tree illustrating the relationships of SsBV3/SZ-150 with selected viruses from *Totiviridae*, *Chrysoviridae*, *Partitiviridae*, *Reoviridae*, *Megabirnaviridae*, *Botybirnavirius*, and unassigned viruses, with the model of VT+G+F. The scale bars correspond to 0.5 amino acid substitutions per site, numbers at branch nodes show percentage posterior probabilities. The red star indicates the position of SsBV3/SZ-150. The detailed information on the selected viruses in the phylogenetic tree is shown in Supplementary Table S1.

The SsBV3/SZ-150 genome is composed of two linear dsRNA segments (L1-dsRNA and L2-dsRNA) of 6,212 and 5,880 bp in length, respectively (Figures 1A, 4C,E). SsBV3/SZ-150 was subjected to BLAST searches against NCBI GenBank, and results showed that SsBV3/SZ-150 shares 96% sequence identity with Botrytis porri botybirnavirus 1 (BpBV1/GarlicBc-72; Wu et al., 2012). Similar to BpBV1, each dsRNA segment of SsBV3 possesses a large open reading frame (ORF), designated as ORF I (on L1-dsRNA), and ORF II (on L2-dsRNA) (Figures 1A, 4C,E). The protein encoded by the 3′ proximal coding region of ORF I encompasses the RdRp_4 conserved domain sequence and shows sequence similarity (97.58%) to the corresponding region of BpBV1/GarlicBc-72. The hypothetical protein encoded by ORF II shares 97.30% identity with BpBV1/GarlicBc-72. In addition, the proteins encoded by the 5′-proximal region of ORF I and by the entire ORF II lack significant sequence similarity to the proteins of any other known virus groups aside from botybirnaviruses. The phylogenetic analysis confirmed that SsBV3/SZ-150 is most closely related to BpBV1/GarlicBc-72 but distant from two other botybirnaviruses reported from *S. sclerotiorum* (Figure 1B). All the above results suggested that SsBV3/SZ-150 and BpBV1/GarlicBc-72 belong to the same species.

#### Characterization of SsMTV1/SZ-150

**FIGURE 2:**
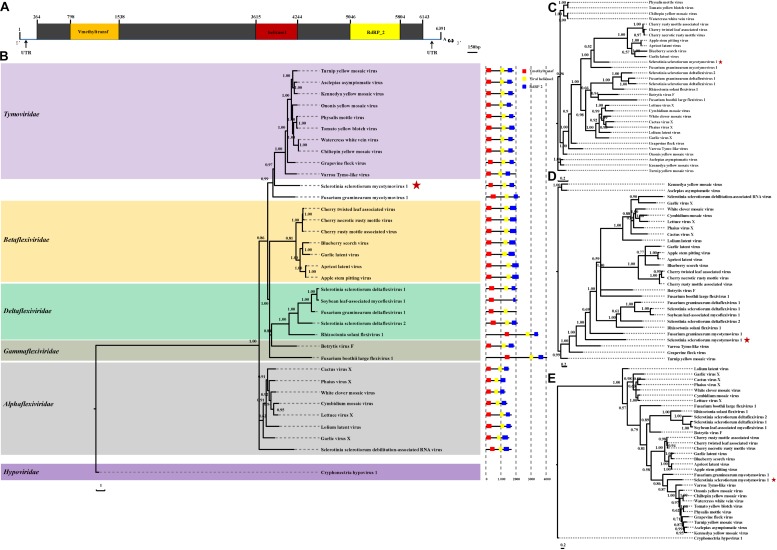
Analyses of identified Sclerotinia sclerotiorum mycotymovirus 1 (SsMTV1/SZ-150) genome. **(A)** Schematic diagrams of the genetic organization of SsMTV1/SZ-150. The ORF (nt positions 264–6,143) encodes a putative viral protein with 1,958 amino acid residues that contains three conserved domains represented by the box: Vmethyltransf (798–1,538 nt), helicase (3,615–4,244 nt), and RNA-dependent RNA polymerase (RdRP_2, 5,046–5,804 nt). 5′-UTR (nt positions 1–263 nt) and 3′-UTR (nt positions 6,144–6,391 nt) are indicated as lines. The scale bar corresponds to a length of 150 bp. Phylogenetic (Bayesian) trees were constructed from the sequences of three conserved domains of the entire RP domain **(B)**, helicase **(C)**, methyltransferase **(D)**, RdRP **(E)**, and the SsMTV1/SZ-150, with the models of VT+I+G+F, RtREV+I+G+F, LG+I+G+F, and LG+I+G+F, respectively. CHV1 (Cryphonectria hypovirus 1) served as an outgroup in the phylogenetic analysis. The scale bar indicates the number of amino acid substitutions per site; numbers at branch nodes show percentage posterior probabilities. The position of SsMTV1/SZ-150 is shown by red asterisks. The detailed information on the selected viruses in the phylogenetic tree is presented in Table 1.

The complete genomic sequence of SsMTV1/SZ-150 is 6391 nt in length excluding the 3′-terminal poly (A) tail (Figures 2A, 4A), with a G+C content of 53.3%. This mycovirus contains a 263- and 248-nt-long 5′- and 3′-UTRs, respectively. Sequence analyses revealed that the SsMTV1 genome has a putative single large ORF. This ORF is 5,880 nt long, beginning at AUG (nt positions 264–266) and terminating at UAG (nt positions 6,141–6,143). The ORF codes for a putative RP of 1,958 amino acids residues with a calculated molecular mass of 218.6 kDa. A conserved-motif search revealed that the RP contains three conserved domains (from the N terminus to the C terminus): viral methyltransferase (Mtr), viral RNA helicase (Hel), and RNA-dependent RNA polymerase (RdRP) (Figures 2A, 4A).

Multiple-alignment analysis was conducted based on the full sequence of RP by the methods of pairwise comparisons and the percentage of amino acid sequence identities. The RP of SsMTV1/SZ-150 showed the highest identity (20.3%) with that of turnip yellow mosaic virus (NP_663297) compared to the RP of the representatives of the order *Tymovirales* (Table 1). Phylogenetic analyses of the viral RP of SsMTV1/SZ-150 and representatives of the order *Tymovirales* indicated that SsMTV1/SZ-150 is phylogenetically related to members of *Tymoviridae* but belongs to a distinct evolutionary lineage (Figure 2B).

**TABLE 1:** Percent amino acid sequence identity between the RP polyprotein, methyltransferase (Mtr), helicase (Hel), and RdRp motifs of Sclerotinia sclerotiorum mycotymovirus 1 (SsMTV1/SZ-150) and those of selected viruses of the family *Tymoviridae*.

**Virus name**	**Accession number**	**RP**	**Mtr**	**Hel**	**RdRp**
Physalis mottle virus	NP_619756	297/1,751 (17.0%)	82/280 (29.3%)	55/203 (27.1%)	100/253 (39.5%)
Tomato yellow blotch virus	AEP40395	305/1,660 (18.4%)	61/178 (34.3%)	57/206 (27.7%)	99/253 (39.1%)
Watercress white vein virus	AFC95826	312/1,670 (18.7%)	67/193 (34.7%)	66/217 (30.4%)	101/253 (39.9%)
Chiltepin yellow mosaic virus	YP_003620401	307/1,670 (18.4%)	67/193 (34.7%)	66/217 (30.4%)	99/253 (39.1%)
Ononis yellow mosaic virus	NP_041257	332/1,707 (19.5%)	73/232 (31.5%)	70/204 (34.3%)	102/253 (40.3%)
Turnip yellow mosaic virus	NP_663297	338/1,667 (20.3%)	77/260 (29.6%)	61/204 (29.9%)	105/254 (41.3%)
Asclepias asymptomatic virus	YP_004464924	266/1,743 (15.3%)	78/252 (31.0%)	63/206 (30.6%)	100/253 (39.5%)
Kennedya yellow mosaic virus	NP_044328	286/1,808 (15.8%)	76/256 (29.7%)	63/202 (31.2%)	101/253 (39.9%)
Grapevine fleck virus	NP_542612	330/1,707 (19.3%)	75/249 (30.1%)	73/203 (36.0%)	89/253 (35.2%)
Varro Tymo-like virus	YP_009159826	329/1,641 (20.1%)	75/239 (31.3%)	51/153 (33.3%)	100/254 (39.4%)
Fusarium graminearum mycotymovirus 1	KT360947	324/1,861 (17.4%)	65/229 (28.4%)	73/208 (35.1%)	97/254 (38.2%)

Multiple-alignment and phylogenetic analyses were next conducted based on three conserved domains (Mtr, Hel, and RdRp) of RP encoded by SsMTV1/SZ-150 (Figures 2C–E). The putative Mtr domain (nt positions 798–1,538) is located near the N-terminal region of the SsMTV1-encoded RP and contains three conserved motifs (I–III; Supplementary Figure S1). Multiple alignment suggested that this conserved domain shared 34.7% sequence identity with those of watercress white vein virus (AFC95826) and chiltepin yellow mosaic virus (YP_003620401; Table 1). The putative Hel domain (nt positions 3,615–4,244) with five conserved motifs (I–V; Supplementary Figure S1) shares 36.0 and 35.1% sequence identities with those of grapevine fleck virus (NP_542612) and Fusarium graminearum mycotymovirus 1 (KT360947), respectively (Table 1). An RdRP domain, containing six conserved motifs (I–VI; Supplementary Figure S1) was detected near the C-terminus of the RP. This domain of SsMTV1 shares highest level of sequence identity (41.3%) with that of turnip yellow mosaic virus (NP_663297; Table 1).

### Analysis of sRNAs Derived From SsMTV1/SZ-150 and SsBV3/SZ-150

**FIGURE 3:**
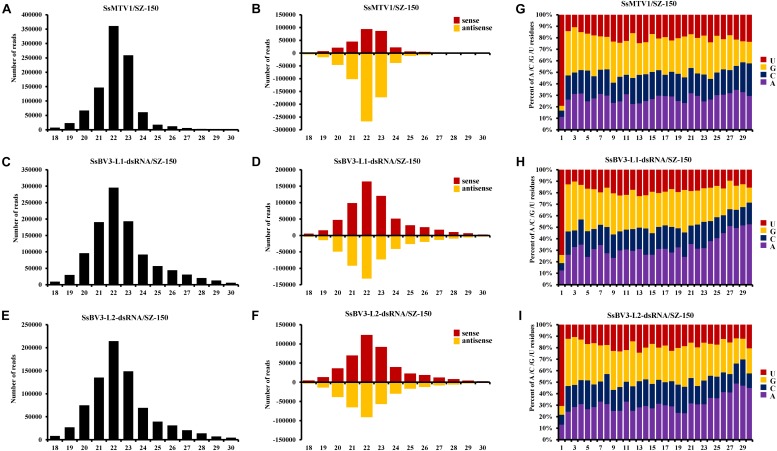
Characterization of vsRNAs from Sclerotinia sclerotiorum mycotymovirus 1 (SsMTV1/SZ-150) and Sclerotinia sclerotiorum botybirnavirus 3 (SsBV3/SZ-150) in mycovirus-infected strain SZ-150. The size distributions of vsRNAs derived from SsMTV1 **(A)**, SsBV3-L1-dsRNA **(C)**, and SsBV3-L2-dsRNA **(E)** in strain SZ-150. The histogram illustrating the number of vsRNA reads; the size distribution of clones 18 to 30 nt. Relative accumulation of vsRNAs from sense and antisense of SsMTV1 **(B)**, SsBV3-L1-dsRNA **(D)**, and SsBV3-L2-dsRNA **(F)** from strain SZ-150. Red and yellow bars indicate sense and antisense vsRNAs, respectively. The identities and the relative frequency of 5′-terminal nucleotide of SsMTV1-derived vsRNAs **(G)**, SsBV3-L1-dsRNA-derived vsRNAs **(H)**, and SsBV3-L2-dsRNA-derived vsRNAs **(I)**. The *y*-axis shows the percentages of 5’-terminal nucleotides consisting of G, C, A, and U in the 18- to 30-nt vsRNAs class, and the *x*-axis represents the length distribution.

In total, 966,210 virus-derived sRNAs (vsRNAs) of 18–30 nt in length, accounting for 7.43% of all sRNA reads, matched the genome sequence of SsMTV1/SZ-150 (Table 2). In addition, 1,077,449 and 794,152 vsRNAs of the same length (accounting for 8.23 and 6.11% of all sRNA reads) matched the genome sequence of SsBV3-L1-dsRNA/SZ-150 and SsBV3-L2-dsRNA/SZ-150, respectively (Table 2). The length of these vsRNAs mainly ranges from 20 to 24 nt, with the 22-nt class being dominant in strain SZ-150. Indeed, the 22-nt class represented approximately 37.3% of the total SsMTV1-derived vsRNAs (Figure 3A and Table 2) and approximately 27.4 and 27.0% of the total SsBV3 vsRNAs (Figures 3C,E and Table 2). The second most abundant vsRNA species in SsMTV1/SZ-150 was the 23-nt class, accounting for 26.8% of the total vsRNAs (Figure 3A and Table 2), whereas the number of vsRNAs (21 and 23 species) was essentially equal in SsBV3/SZ-150 (Figures 3C,E and Table 2). A more specific analysis was performed on the distribution of the vsRNAs derived from the sense and antisense strands of SsMTV1. The results showed that there was an asymmetrical distribution, and the antisense vsRNAs were dominant (nearly 70%; Figure 3B and Table 2). In contrast to the vsRNAs from SsMTV1/SZ-150, the number of vsRNAs derived from the sense strand was almost equal to that from the antisense strain in SsBV3/SZ-150 (Figures 3D,F and Table 2).

**TABLE 2:** The number of vsRNAs derived from Sclerotinia sclerotiorum mycotymovirus 1 (SsMTV1/SZ-150) and Sclerotinia sclerotiorum botybirnavirus 3 (SsBV3/SZ-150).

**Virus**	**Total**		**20nts**		**21nts**		**22nts**		**23nts**		**24nts**	
	**Sense**	**Anti-sense**	**Sense**	**Anti-sense**	**Sense**	**Anti-sense**	**Sense**	**Anti-sense**	**Sense**	**Anti-sense**	**Sense**	**Antisense**
SsMTV1/SZ-150	295,150	671,063	21,094	46,132	44,906	101,794	93,859	266,997	86,258	172,831	22,404	38,368
SsBV3-L1-dsRNA/SZ-150	596,387	481,062	47,154	48,948	98,526	91,967	164,287	131,221	120,287	72,818	51,181	40,751
SsBV3-L2-dsRNA/SZ-150	447,227	346,925	36,374	38,468	69,877	65,239	123,340	91,039	91,892	56,901	39,443	29,826

*Sense indicates the vsRNAs derived from the sense strand of virus. Antisense indicates the vsRNAs derived from the antisense strand of virus.*

**FIGURE 4:**
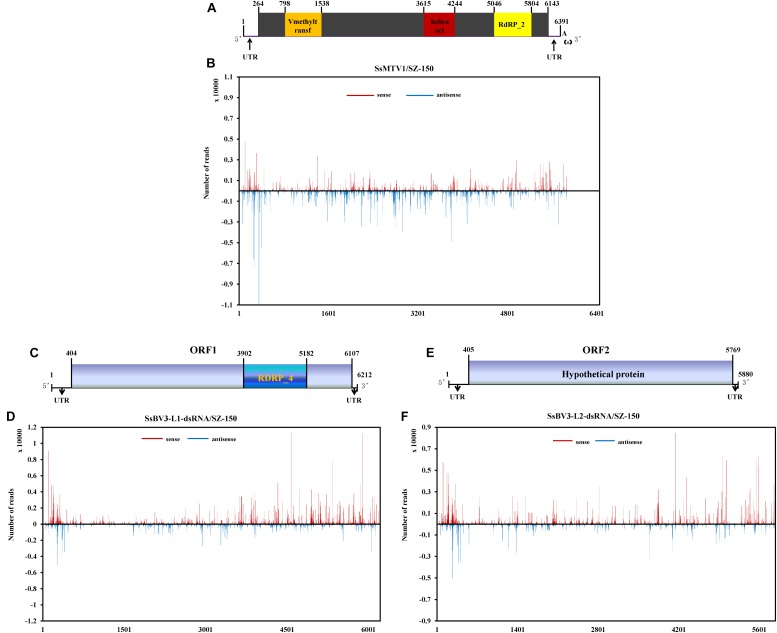
Profiles of vsRNAs along Sclerotinia sclerotiorum mycotymovirus 1 (SsMTV1/SZ-150) and Sclerotinia sclerotiorum botybirnavirus 3 (SsBV3/SZ-150) genomes. The identified vsRNAs were mapped onto the SsMTV1 **(A)**, SsBV3-L1-dsRNA **(C)**, and SsBV3-L2-dsRNA **(E)** genomes. **(B**,**D**,**F)** Distribution of the vsRNAs mapped on the corresponding viral genomes. The *x*-axis shows schematic representations of SsMTV1 and SsBV3 genomic organization. The *y*-axis indicates the numbers of vsRNAs matching the sense strand (red, above the *x*-axis) and antisense strand (blue, below the *x*-axis).

There was a clear preference for uridine (U) residues (79.2%) in the 5′ terminal nucleotide composition of SsMTV1-derived vsRNAs, while guanidine (G) was the least abundant (4.1%; Figure 3G). Similar results were obtained in the analysis of vsRNAs from SsBV3/SZ-150 (Figures 3H,I). To determine the distribution of the vsRNAs in genomes of SsMTV1/SZ-150 and SsBV3/SZ-150, vsRNAs reads were then aligned against the genomes of two mycoviruses. As presented in Figures 4B,D,F, vsRNAs were distributed along the whole viral genome, including the coding regions and 5′- or 3′-UTRs; the vsRNAs derived from SsMTV1/SZ-150 were distributed along the genome in a non-random pattern, with the majority derived from the negative strand, and the vsRNAs derived from SsBV3/SZ-150 were uniformly distributed along the positive and negative strands, as described previously. There was one hotspot region, which produced more vsRNAs; they were present on both the positive and negative strands in the 5′ region of genomes of SsMTV1/SZ-150 and SsBV3/SZ-150.

### SsBV3/SZ-150 and SsMTV1/SZ-150 Make Only a Limited Contribution to the Hypovirulence of *S. sclerotiorum*

**FIGURE 5:**
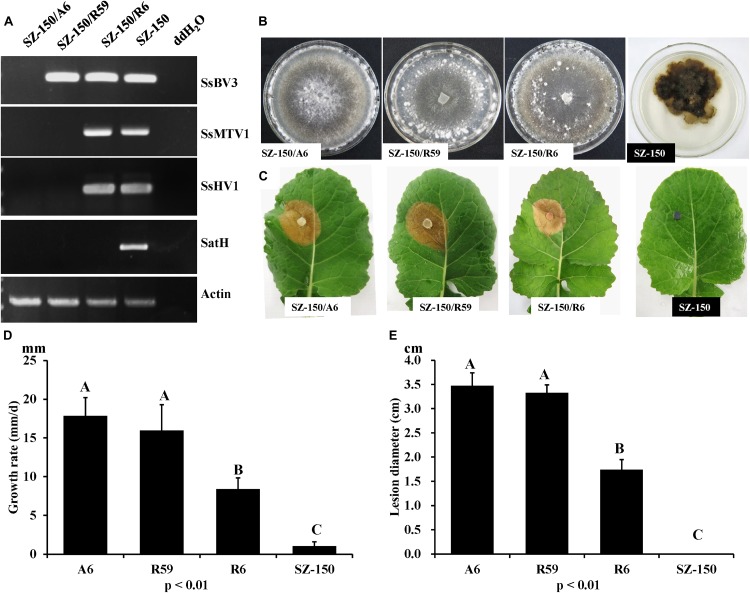
Biological properties of four individual strains: SZ-150/A6, SZ-150/R59, SZ-150/R6, and SZ-150. **(A)** Detection of mycoviruses Sclerotinia sclerotiorum hypovirus 1 (SsHV1/SZ-150) and its related satellite RNA (SatH), Sclerotinia sclerotiorum mycotymovirus 1 (SsMTV1/SZ-150), and Sclerotinia sclerotiorum botybirnavirus 3 (SsBV3/SZ-150) in four strains by RT-PCR. The actin gene served as an internal control. **(B)** Colony morphology and **(D)** growth rate of four individual strains. Four strains were grown on PDA for 15 days at 20^∘^C prior to photography. **(C,E)** Virulence assays of the four strains on the detached leaves of rapeseed plants. Statistical analysis of the growth rate and lesion diameter of the four individual strains was conducted at the *P* < 0.01 level. The error bars indicate the SD from three sample means.

RT-PCR analysis was performed on total-RNA samples from mycelial extracts of the hypovirulent strain SZ-150, from an ascospore progeny derivative SZ-150/A6, and from two protoplast derivatives (SZ-150/R59 and SZ-150/R6) of strain SZ-150. Strain SZ-150/R59 was infected with SsBV3/SZ-150, while SZ-150/R6 was coinfected by three mycoviruses: SsMTV1/SZ-150, SsHV1/SZ-150, and SsBV3/SZ-150 (Figure 5A). Strain SZ-150/A6 was virus-free, while we failed to create a strain infected with SsMTV1 alone (Figure 5A). To determine the biological effects of SsBV3/SZ-150 and SsMTV1/SZ-150 on hypovirulence of *S. sclerotiorum*, biological features of the four individual strains were assayed. Strain SZ-150 had abnormal colony morphology characterized by a dark pigment, whereas its derivatives SZ-150/A6, SZ-150/R59, and SZ-150/R6 had normal colony morphology with no significant differences on the potato–dextrose–agar (PDA) medium (Figure 5B). The growth rate of SZ-150/R59 (15.97 ± 3.34 mm/day) was not significantly different from that of SZ-150/A6 (17.86 ± 2.33 mm/day), but was faster than that of SZ-150/R6 (8.40 ± 1.46 mm/day; Figure 5D). Virulence assays were conducted on the detached leaves of rapeseed plants that were kept at 20°C for 48 h post inoculation. Strain SZ-150 failed to infect rapeseed leaves, whereas SZ-150/A6, SZ-150/R59, and SZ-150/R6 induced typical lesions on the leaves (Figure 5C). The size of lesion induced by SZ-150/R59 (3.3 ± 0.17 cm) was not significantly different from that induced by strain SZ-150/A6 (3.48 ± 0.26 cm) but was larger than that of strain SZ-150/R6 (1.74 ± 0.21 cm; Figure 5E). These results suggest that SsBV3/SZ-150 does not induce phenotypic changes in the host, and SsMTV1/SZ-150 makes only a limited contribution to the hypovirulence.

## Discussion

Multiple infection by several mycoviruses is a common phenomenon in the fungi kingdom (Ghabrial and Suzuki, 2008). These viruses are usually detected by conventionally methods, such as dsRNA extraction. Nevertheless, hypovirulence-related mycoviruses receive more attention from researchers, while latent mycoviruses or mycoviruses with a lower copy number of dsRNA formed in a fungal host are often ignored or are difficult to detect by dsRNA extraction.

Previously, our study based on the dsRNA extraction and conventional cloning strategies revealed coinfection by two mycoviruses SsHV1/SZ-150 and SsBV3/SZ-150 (SsRV1/SZ-150 in Xie et al., 2011) in a hypovirulent strain SZ-150 of *S. sclerotiorum*. In the present study, the results of high-throughput sequencing of sRNAs revealed one additional mycovirus (SsMTV1/SZ-150) in the same strain and, combined with RACE, allowed for completion of genome sequences of two mycoviruses (SsMTV1/SZ-150 and SsBV3/SZ-150). High-throughput sequencing is a powerful tool for the discovery of many novel ssRNA mycoviruses, especially (−)ssRNA mycoviruses that have a lower copy number of dsRNA in fungal cells. Several studies have proved the ability of high-throughput sequencing to detect low-copy mycovirus infections, thereby discovering new mycoviruses and evaluating mycovirus diversity in phytopathogenic fungi including *S. sclerotiorum*, *Rhizoctonia solani*, *Rosellinia necatrix*, and *Rhizophagus* spp. (Bartholomaus et al., 2016; Marzano et al., 2016; Yaegashi et al., 2016; Mu et al., 2017).

The genome of SsBV3/SZ-150 shares 97% identity with that of BpBV1/GarlicBc-72 at the amino acid level, thus revealing that SsBV3/SZ-150 should be recognized as an isolate of species Botrytis porri botybirnavirus 1. BpBV1/GarlicBc-72 confers hypovirulence on its natural host *B. porri* (Wu et al., 2012), whereas SsBV3/SZ-150 is associated with a latent infection in *S. sclerotiorum*, suggesting that BpBV1 patterns of interaction with *B. porri* and *S. sclerotiorum* are different, even though these two fungi share 83% amino acid identity in the genome (Amselem et al., 2011). Actually, two mycoviruses of SsBV1 and SsBV2 belonging to the genus *Botybirnavirus* have already been characterized from *S. sclerotiorum* (Liu et al., 2015; Ran et al., 2016). SsBV2 infection induces hypovirulence in strains of *S. sclerotiorum* studied. Although infection by SsBV1 alone has no significant effects on culture morphology and virulence of *S. sclerotiorum*, infection by SsBV1 along with its SatH leads to slightly reduced virulence and a slower mycelial growth rate in its host. These combined results imply that the interaction between botybirnavirus and *S. sclerotiorum* is complex. Botybirnaviruses probably have a wide host range. Besides *Botrytis* spp. and *S. sclerotiorum*, botybirnaviruses have also been found in the fungi *Bipolaris maydis* and *Alternaria* spp. (Xiang et al., 2017; Wang et al., 2018). Although the genomes of these two botybirnaviruses have been fully sequenced, the influences on their fungal hosts are still unknown.

Notably, a botybirnavirus, BpBV1, was detected in both *Botrytis squamosa* and *B. cinerea* in addition to *B. porri* and *S. sclerotiorum*. These four phytopathogenic fungi have many similarities in the genome; more importantly, they have similar ecological niches and have opportunities to coinfect the same plants. Similar findings have been reported in other studies. Some viruses detected in *R. necatrix* have the closest relation to viruses infecting *Fusarium* spp., which are sympatric to *R. necatrix* (Arjona-Lopez et al., 2018). Recently, Schoebel et al. (2018) provided evidence that multiple interspecific virus transfers have occurred from *Hymenoscyphus albidus* to *Hymenoscyphus fraxineus* (Schoebel et al., 2018). These data suggest that interspecific transmission, even interfamily transmission, of BpBV1, and other mycoviruses may occur under field conditions, although the underlying mechanism is still unclear.

The complete genome sequence of SsMTV1/SZ-150 was determined in this study as well. Phylogenetic analyses of the putative polyprotein strongly suggest that SsMTV1/SZ-150 is related, albeit distantly, to members of the family *Tymoviridae* and forms an independent evolutionary clade. Therefore, SsMTV1/SZ-150 is a novel mycotymo-like virus related to members of *Tymoviridae*. Although most viruses in the order *Tymovirales* infect plants, a few members of this order infecting plant-pathogenic fungi, including *B. cinerea*, *Fusarium boothii*, *Fusarium graminearum*, *R. solani*, and *S. sclerotiorum*, have been characterized (Howitt et al., 2006; Li K. et al., 2016; Li P. et al., 2016; Bartholomaus et al., 2017; Mizutani et al., 2018). Nevertheless, those mycotymo-like viruses were placed into different evolutionary lineages, suggesting that mycotymo-like viruses are more diverse in fungi than previously thought, and have a complex evolutionary relationship with plant viruses within *Tymovirales*.

The genomic RNA of viruses belonging to the family *Tymoviridae* ranges from 6.0 to 7.5 kb in length and is mostly polyadenylated (King et al., 2011) and characterized by a high cytosine content (32–50%). Similar to other members of family *Tymoviridae*, the genomic sequence of SsMTV1/SZ-150 is 6,391 nt in size, excluding the 3′-terminal poly (A) tail. Nevertheless, the genomic sequence of SsMTV1/SZ-150 also contains a high content of cytosines with 30.1%. Consistently with the genomic RNA of Sclerotinia sclerotiorum debilitation-associated RNA virus (Xie et al., 2006), the genome of SsMTV1/SZ-150 has a single ORF encoding a polyprotein but lacks a gene encoding a coat protein (CP). All other recognized members of the order *Tymovirales* contain CP genes regardless of whether they are plant viruses, insect viruses, or two mycoviruses (BotVX and BotV-F).

In the present study, we analyzed the vsRNAs derived from SsMTV1/SZ-150 and SsBV3/SZ-150. vsRNAs from SsMTV1/SZ-150 and SsBV3/SZ-150 have a size from 18 to 30 nt with a peak at 22 nt. vsRNAs are uniformly distributed along the genomes of two viruses, with the majority derived from the negative strand in the case of SsMTV1/SZ-150. In the plant *Arabidopsis thaliana*, different Dicer proteins are responsible for the cleavage of sRNAs of different lengths. Dicer2 is responsible for the cleavage of 22-nt sRNAs (Ruiz-Ferrer and Voinnet, 2009), suggesting that the homolog of DCL2 in *S. sclerotiorum* might be the predominant Dicer ribonuclease involved in the biogenesis of vsRNAs. Some reports indicate that the loading of sRNA onto an AGO-containing effector complex is guided by the 5′-terminal nucleotide of the sRNA in *Arabidopsis* and rice (Mi et al., 2008; Wu et al., 2009). In *Arabidopsis*, AGO1 harbors microRNAs with 5′-terminal U residues (Mi et al., 2008). This finding suggests that most of the vsRNAs are preferentially recruited into AGO1 in *S. sclerotiorum*.

Thus, aside from SsHV1/SZ-150 and its SatH, in this study, we discovered and characterized two other mycoviruses, SsBV3/SZ-150 (belonging to genus *Botybirnavirius*), and SsMTV1/SZ-150 (related to viruses in the order *Tymovirales*), in the hypovirulent strain SZ-150 *via* high-throughput sequencing. The SsMTV1 and SsBV3-vsRNA analyses suggest that the DCL2 and AGO1 are key players in the defense against mycovirus infection in *S. sclerotiorum*.

## Materials and Methods

### Fungal Strains and Culture Conditions

*Sclerotinia sclerotiorum* strain SZ-150 was originally isolated from a sclerotium collected from a diseased rapeseed (*Brassica napus*) (Xie et al., 2011). SZ-150/R59 and SZ-150/R6 were derived from strain SZ-150 by protoplast isolation and regeneration. SZ-150/A6 was a single-ascospore isolation of strain SZ-150/R59. All fungal strains were grown at 18–22°C on the PDA medium and stored on PDA slants at 4–8°C.

### sRNA Sequencing and Bioinformatics Analysis

To prepare total RNA for high-throughput sRNA sequencing, strain SZ-150 was cultured on cellophane membranes overloading PDA plate for 10 days. The harvested mycelium of SZ-150 was ground into a fine powder in liquid nitrogen with a mortar and pestle, and total RNA was extracted with TRIzol RNA extraction kit (Takara Bio, Inc., Japan). The total RNA was stored at −80°C until analysis. Deep sequencing of sRNA was performed on the Illumina HiSeq 2000 platform by the BGI Tech Company (Shenzhen, China). sRNA molecules (≤30 nt) were isolated and purified by polyacrylamide gel electrophoresis (PAGE). The purified sRNA was ligated with an adaptor, and then reverse-transcribed to cDNA, which was then amplified by polymerase chain reaction (PCR) and recovered by PAGE in a 6% gel for deep sequencing. To obtain clean sequences, the raw reads from deep sequencing were processed to remove the adaptor sequences and discard low-quality reads, and then were assembled into contigs in the Velvet software with a k-mer value of 17 excluding the sRNA generated from *S. sclerotiorum*. The assembled contigs were employed in searches in the GenBank database^2^ using BLASTN and BLASTX to find similar sequences.

Reads of vsRNAs were mapped to the virus genome using the Bowtie (1.0) software and only those having sequences identical or complementary to the viral genomic sequence (allowing for one mismatch with the reference genomes) were identified as vsRNAs. Then, the sorted and indexed BAM file was processed by viRome^3^.

### Full-Length cDNA Cloning, Sequencing, Sequence Analysis, and Phylogenetic Analysis

To fill the gap between different contigs, virus-specific primers based on the sequences assembled from sRNAs were designed and used for RT-PCR (Supplementary Table S2). The amplicons were purified and then cloned into the pMD18-T vector (Takara, Dalian, China) for sequencing. Finally, we filled the gap between two different contigs. To obtain the complete cDNA sequences of mycovirus genomes, the terminal sequence was determined by RACE-PCR using the SMARTer RACE 5′/3′ Kit (Cat. No. 634858, Clontech) (Supplementary Table S2).

Sequence assembly and the basic features (e.g., lengths, G+C content, and ORF identification) of the full-length genome sequence were analyzed using the DNAMAN software. The conserved domains of mycovirus genomes were identified on a motif scan website^4^. The sequences of previously reported mycoviruses and other sequences referenced in this research were downloaded from the NCBI GenBank database^5^ and used for multiple-alignment and phylogenetic analyses. Multiple alignment was performed using the Clustal X program. On the basis of the aligned sequences, the best-fit evolution models of SsMTV1 and SsBV3 were obtained using Akaike’s information criterion (AIC), and searched using the ProtTest server. BI (Bayesian) trees were constructed with MrBayes-3.2.7, and then the tree files were viewed using FigTree-1.4.0.

### Assay of Biological Properties of Mycovirus-Infected and Mycovirus-Free Strains

To determine the contribution of SsMTV1 and SsBV3 to the hypovirulence in strain SZ-150, the presence of viruses in strains SZ-150, SZ-150/A6, SZ-150/R59, and SZ-150/R6 was analyzed *via* RT-PCR using specific primers for SsHV1, SsMTV1, SsBV3, and SatH (Supplementary Table S2). Four strains, SZ-150, SZ-150/A6, SZ-150/R59, and SZ-150/R6, were evaluated regarding colony morphology, growth rate, and virulence as previously described (Zhang et al., 2009; Xie et al., 2011). All assays were repeated three times. Experimental data were analyzed in SAS 8.0 software. Treatment means were compared by the least significant difference test at *P*n SAS 8.

## Data Availability

All datasets generated for this manuscript can be found in GenBank. Accession numbers are listed in the Supplementary Information.

## Author Contributions

QW, DJ, and JX designed the research and wrote the manuscript. QW, SC, and XX executed the experiments. QW, XX, JC, YF, TC, DJ, and JX performed the data and bioinformatics analyses. All authors read and approved the final manuscript.

## Conflict of Interest Statement

The authors declare that the research was conducted in the absence of any commercial or financial relationships that could be construed as a potential conflict of interest.

## Footnotes

1 https://talk.ictvonline.org/taxonomy

2 http://www.ncbi.nlm.nih.gov/

3 http://sourceforge.net/projects/virome

4 http://www.genome.jp/tools/motif/

5 http://www.ncbi.nlm.nih.gov/genomes
