# Supplementary material for: Discovery of Two Mycoviruses by High-Throughput Sequencing and Assembly of Mycovirus-Derived Small Silencing RNAs From a Hypovirulent Strain of Sclerotinia sclerotiorum
Source: Front Microbiol. 2019 Jul 2;10:1415. doi: 10.3389/fmicb.2019.01415 (PMC6626909; doi:10.3389/fmicb.2019.01415)
Supplement: Supplementary file 1 [file Data_Sheet_1.pdf]

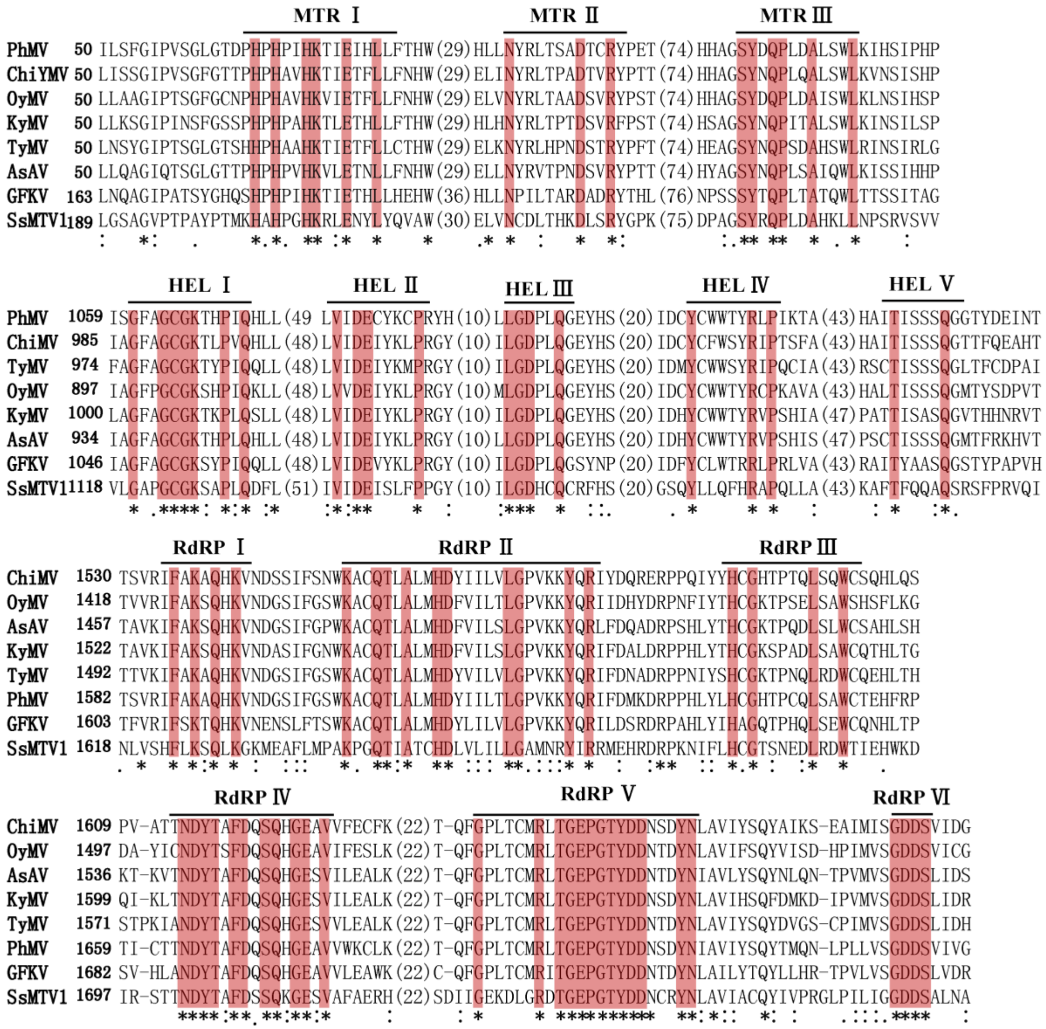
MTR Ⅰ
MTR Ⅱ
MTR Ⅲ
HEL Ⅰ
HEL Ⅱ
HEL Ⅲ
HEL Ⅳ
HEL Ⅴ
RdRP Ⅰ
RdRP Ⅱ
RdRP Ⅲ
RdRP Ⅳ
RdRP Ⅴ
RdRP Ⅵ
PhMV
ChiYMV
OyMV
KyMV
TyMV
AsAV
GFKV
SsMTV1
ChiMV

Figure S1. Multiple alignment of three conserved domains (Mtr, Hel, and RdRp) of Sclerotinia sclerotiorum mycotymovirus 1 (SsMTV1/SZ-150) with corresponding regions in the referenced members of the order *Tymovirales*. The positions of the motifs are indicated by lines above the sequences with numbers from I to VI. Identical residues are shaded and indicated by asterisks; conserved and semi-conserved amino acid residues are indicated by colons and dots, respectively. Numbers in square brackets correspond to the number of amino acid residues separating the motifs. ChiYMV: Chiltepin yellow mosaic virus, OYMV: Ononis yellow mosaic virus, AsAV: Asclepias asymptomatic virus, KyMV: Kennedya yellow mosaic virus, TYMV: Turnip yellow mosaic virus, PhyMV: Physalis mottle virus, GFkV: Grapevine fleck virus.

Table S1 The information of all selected viruses used for phylogenetic analysis

Virus Family	Virus name	Abbreviation	Accession Numbe
Chrysovidae	**Colletotrichum gloeosporioides chrysovirus 1**	**CgCV1**	**KT581957**
	**Raphanus sativus chrysovirus 1**	**RsCV1**	**JQ045335**
	**Botryosphaeria dothidea chrysovirus 1**	**BdCV1**	**KF688736**
	**Fusarium graminearum dsRNA mycovirus-2**	**FgV2**	**HQ343295**
	**Magnaporthe oryzae chrysovirus 1-A**	**MoCV-1A**	**AB560761**
	**Macrophomina phaseolina chrysovirus 1**	**MpCV1**	**KP900886**

	**Verticillium dahliae chrysovirus 1**	**VdCV1**	**NC_038784**
Totiviridae	**Saccharomyces cerevisiae virus L-A**	**ScV-L-A**	**J04692**
	**Saccharomyces cerevisiae virus L-BC**	**ScV-L-BC**	**U01060**
	**Magnaporthe oryzae virus 2**	**MoV2**	**AB300379**
	**Gremmeniella abietina RNA virus L1**	**GaRV-L1**	**AF337175**
	**Botryotinia fuckeliana totivirus 1**	**BfTV1**	**AM491608**
Partitiviridae	**Helicobasidium mompa partitivirus V1-1**	**HmV-V1-1**	**AB110979**
	**Rosellinia necatrix virus 1**	**RnV1**	**AB113347**
	**Rhizoctonia solani virus 717**	**RhsV-717**	**AF133290**
	**Rosellinia necatrix partitivirus 2**	**RnPV2**	**NC_020234**
	**Rhizoctonia solani dsRNA virus 2**	**RsPV2**	**NC_023684**
	**Sclerotinia sclerotiorum partitivirus S**	**SsPV-S**	**NC_013014**
	**Sclerotinia sclerotiorum partitivirus 1**	**SsPV1**	**JX297511**
Reoviridae	**Cryphonectria parasitica mycoreovirus 1**	**CpMYRV1**	**NC_010743**
	**Rosellinia necatrix mycoreovirus 3**	**RnMYRV3**	**NC_007535**
	**Sclerotinia sclerotiorum mycoreovirus 3**	**SsMYRV4**	**NC_030158**
Megabirnaviridae	**Pleosporales megabirnavirus 1**	**PMBV1**	**KT601119**

	**Rosellinia necatrix megabirnavirus 1**	**RnMBV1**	**NC_013462**
	**Sclerotinia sclerotiorum megabirnavirus 1**	**SsMBV1**	**NC_027221**
	**Rosellinia necatrix megabirnavirus 2**	**RnMBV2**	**NC_029125**
Botybirnaviridae	**Sclerotinia sclerotiorum botybirnavirus 1**	**SsBV1**	**NC_027138**
	**Sclerotinia sclerotiorum botybirnavirus 2**	**SsBV2**	**KT962972**
	**Bipolaris maydis botybirnavirus 1**	**BmBRV1**	**MF034087**
	**Alternatra botybirnavirus 1**	**ABRV1**	**KX784491**
	**Soybean leaf-associated botybirnavirus 1**	**SlaBRV1**	**KT598241**
	**Botrytis porri botybirnavirus 1**	**BpBV1**	**NC_017990**
**Unclassified**	**Fusarium graminearum virus DK21**	**FgV-DK21**	**AY533037**
	**Sclerotinia sclerotiorum RNA virus L**	**SsRV-L**	**EU779934**
	**Sclerophthora macrospora virus A**	**SmV-A**	**AB083060**

Table S2 Primer pairs used in this study.

Primer name	Primer sequence (5′–3′)	Application	Product size (bp)
actin-qF2	GAGCTGTTTTCCCTTCCATTGTC	Used as an internal control	146
actin-qR4	GACGACACCGTGCTCGATTGG		
SsHV1-F	CCAAAACAACCCTGTCGCC	Detection for SsHV1	252
SsHV1-R	CAACAAACCTGTCAGCACCC		
SsBV3-F	GGGTGACGACGGATGGGTA	Detection for SsBV3	462
SsBV3-R	CACAGGCAGCTTCGCTACTCT		
SatH-F	AGAAATTGTTATGGCAAGCT	Detection for SatH	1271
SatH-R	GGGTTGATATATCCGATAAG		
SsMTV1-F	ATTGGTCAAGACTCCGTGAC	Detection for SsMTV1	1020
SsMTV1-R	GGAAGCATGCGCCGCTTGGT		
random dN6	CGATCGATCATGATGCAATGCNNNNNN	To obtain the viral cDNA	
Oligo (dT)18	d(TTTTTTTTTTTTTTTTTT)		
5’Long primer	CTAATACGACTCACTATAGGGCAAGCAGTGGTATCAACGCAGAGT	Termini determination of SsMTV1	
RACE-OLIGO:	GCATTGCATCATGATCGATCGAATTCTTTAGTGAGGGTTAATTGCC-(NH2)		
O5RACE -1	GGCAATTAACCCTCACTAAAG		
O5RACE -2	TCACTAAAGAATTCGATCGATC		
O5RACE -3	CGATCGATCATGATGCAATGC		
SsMTV1_5’1	GAAAGGGTCGTAACGGTATTCG		
SsMTV1_5’2	CTGTGGAGATAGCCATCGTTCA		
C1-F	GACGAAATTTCTCTCTTTCCCCCG	To fill the gap of SsMTV1	
C1-R	TATTATACTgTCTAgTTCCgAC		

C2-F	GTAGAACTCTCTGACACCTCAA		
C2-R	GGGCCGCGATCTGACCATGGCAGT		
SsBV3_L1-1F	GCAAATAAAAGAGCTGCCTT	To determine the genome of SsBV3-L1-dsRNA	
SsBV3_L1-1R	ATCTTCTTCTAATACCACCT		
SsBV3_L1-2F	CACCACAAGCGCAGTGACGA		
SsBV3_L1-2R	ATCTTCTTCTAATACCACCT		
SsBV3_L1-3F	TGAGTCAGAGATGGAGGAGA		
SsBV3_L1-3R	CAGGTAGCGTGATCATGTTG		
SsBV3_L1-4F	TGACAGCAGAAGGATTCAAC		
SsBV3_L1-4R	TCTAACCTGTTCTTGCCCAA		
SsBV3_L1-5F	GCAGAAAGTAGCGCAGGAGA		
SsBV3_L1-5R	AAACTCACCTTTCGCCCACT		
SsBV3_L1-6F	GATCAAAGAAGGGATCTTGA		
SsBV3_L1-6R	CTACCCATCCGTCGTCACCC		
SsBV3_L1-7F	AACCTCACGTACCACCACAT		
SsBV3_L1-7R	GCTATCTATTCACTTCTCCC		
SsBV3_L2-1F	GCAAATAAAAGAGCTGCCTA	To determine the genome of SsBV3-L2-dsRNA	
SsBV3_L2-1R	GGCATCTTGTTTGGTTTGAA		
SsBV3_L2-2F	TGTGCATCGCTATCGCGCGC		
SsBV3_L2-2R	AAGTGAATTGCACAACGGTA		
SsBV3_L2-3F	GTTCAGATACAATAAGACAG		
SsBV3_L2-3R	GCAAATCGGTCTGCACAGTG		
SsBV3_L2-4F	CRAAGGCTCGCCCACACGGG		
SsBV3_L2-4R	GGTGTCTTTGCGGGAAAGAG		
SsBV3_L2-5F	TGGCCGAGTTCGGTGAAAGA		

SsBV3_L2-5R	AGCTTCTCTTCTGTACCCGC		
SsBV3_L2-6F	AAAAGGCACAGGAGAGGGAG		
SsBV3_L2-6R	GCTCTGTAATTACTCCTTCC		
